# Patient involvement in cardiovascular research: a qualitative impact evaluation

**DOI:** 10.1186/s40900-019-0165-z

**Published:** 2019-10-14

**Authors:** Eva Vroonland, Inge Schalkers, Daphne Bloemkolk, Christine Dedding

**Affiliations:** 1 Harteraad, Prinses Catharina-Amaliastraat 10, 2496 XD Den Haag, The Netherlands; 2 De Hartstichting, Prinses Catharina-Amaliastraat 10, 2496 XD Den Haag, The Netherlands; 3 Amsterdam UMC, De Boelelaan 1089a, 1081 HV Amsterdam, The Netherlands

**Keywords:** Patient participation, Patient and public involvement, Cardiovascular research, Impact evaluation, Impact on research

## Abstract

**Background & Objective:**

Involving patients in scientific research has been shown to improve the relevance of the research, as well as its quality and applicability. Harteraad, the Dutch patient organization for people with cardiovascular diseases, has a Committee of Experienced Experts (patients) advising researchers on the content of grant proposals prior to submission. Until now, the impact of the committee’s advice was unknown. This study, initiated by Harteraad, aimed to evaluate the impact of the provided advice on the content of grant proposals and investigate how to strengthen this impact.

**Methods:**

Fourteen grant proposals both prior to and after receiving the committee’s advice were compared in order to analyse how the advice had been incorporated into the final proposal. Subsequently, 10 researchers who received the committee’s advice were interviewed. Moreover, a focus group discussion was conducted with five committee members.

**Results:**

Document analysis showed that almost 40% of the advice was incorporated in the final grant proposals. Researchers made several changes to their proposals, such as increasing the extent of patient involvement throughout the research, use of simpler language, and/or adding information on the consequences of an intervention for patients. Advice requiring fundamental changes in the research design was most often not incorporated. This finding was confirmed by the interviewees, although some stressed to use the committee’s advice later on during the execution of the research. According to the interviewees and members of the committee, the impact of the committee’s advice could be strengthened in several ways, including 1) improving training/education for researchers and the committee, 2) organizing dialogues between patients and researchers, 3) aligning perspectives between funding bodies and patient organizations on what is expected from researchers, 4) making it obligatory for the researchers to clarify how the patient’s advice was incorporated, and 5) fostering researchers’ internal motivation for involvement. Committee members have contributed to implementing these recommendations.

**Conclusion:**

The committee’s advice has considerable impact on the content of grant proposals. However, effort is required to increase the value that is currently attributed to patient involvement, and to support researchers in the required organizational and cultural changes to meaningfully involve patients in research.

## Plain English summary

It has come to be acknowledged that clinical and health service research does not always meet patients’ needs and daily realities. Research has shown to become more relevant, applicable, and of higher quality if patients are involved in the research process. The Dutch patient organization for people with cardiovascular diseases (Harteraad) established a Committee of Experienced Patients, who have personal experiences with cardiovascular diseases, to advise researchers on their proposals before submitting these for funding. This study aimed to explore the impact of the patient committee’s advice on the research proposals and how to strengthen this impact. Proposals from before and after the committee’s advice were compared; ten researchers were interviewed, five members of the committee participated in a focus group discussion. This research was commissioned and largely executed by Harteraad.

This study showed that almost 40% of the points of advice given by the committee were incorporated into the final proposals; researchers changed their use of language, added information on intervention consequences, and incorporated ideas for more active patient involvement. The interviewed researchers and committee members had several ideas to strengthen the impact of the committee’s advice, including: 1) more and better training for researchers on why and how to collaborate with patients, 2) organizing dialogues between patients and researchers, 3) making it obligatory for researchers to clarify how they incorporated the patient’s advice. 4) Aligning perspectives between funding bodies and patient organizations on what is expected from researchers, and 5) Fostering researchers’ intrinsic motivation for involvement.

## Background

Over the last decades, patient and public involvement in scientific research has become increasingly important. Involving patients in health research has shown to improve the relevance and suitability of research, as well as the quality and applicability of the research outcomes [1–4]. Furthermore, it has also been shown to enhance the overall legitimacy of scientific research [5–8]. Numerous stakeholders worldwide, such as patient organizations, healthcare professionals, universities, health funds, and research funding bodies recognize the importance and relevance of patient involvement within research [4, 9, 10]. Despite an increasing body of knowledge, formal institutions like healthcare funding bodies and universities still struggle with *how* to involve patients in a meaningful way, in a way that might actually have an impact.

**Fig. 1:**
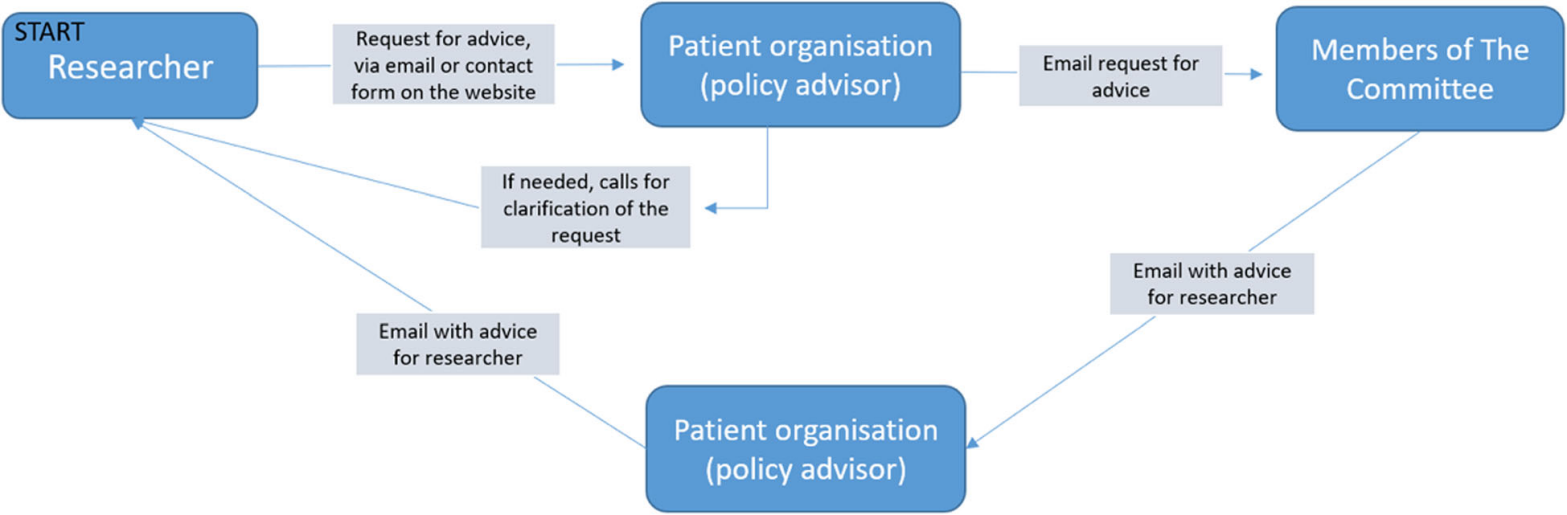
Schematic representation of how Harteraad organizes patient participation in the phase of writing grant proposals

Harteraad, a Dutch patient organization for people with cardiovascular and venous diseases and their loved ones, established a patient committee to facilitate, among other things, patient involvement in designing a grant proposal. Figure 1 shows a schematic representation of this approach. This patient panel was established in 2012. It consists of 12 members at the time of this research and is coordinated by a policy advisor of Harteraad who has particular expertise in the field of PPI. The members of the patient committee all have personal experience with cardiovascular and/or venous diseases. Most of them are highly educated and very motivated to collaborate with scientific researchers. All members followed a two-day training course given by a patient advocate, addressing the theory and practice of patient involvement, including how to critically read and comment on a research proposal from the patient perspective. They currently do not get reimbursement for their advice, but travel expenses, phone costs, and training are covered by Harteraad. Members of the committee advise researchers via email on their original, scientific draft grant proposals (in English) from the patient’s perspective. They provide feedback on six main criteria, which were formulated by members of the committee in 2013. These criteria include relevance, applicability/feasibility, safety, understandable language, patient input, and communication. Moreover, members of the committee can emphasize some general remarks, for instance about what they think is particularly good or bad about the proposal. This approach (as summarized in Fig. 1) is relatively new and unique in the Netherlands. Little was known about how this approach could be organized best. Moreover, it was unknown what the actual impact of the advice has been on the content of grant proposals. Knowing the impact of participatory activities is necessary to justify the required investments of *all* involved, to ensure genuine involvement that goes beyond mere tokenism.

Therefore, Harteraad initiated this study, aiming to evaluate whether or not the committee’s advice helped researchers to improve their applications for funding. We also aimed to investigate how the impact of the advice could be strengthened.

### Theoretical background

Patient involvement is a complex phenomenon without a univocal definition. Referring to similar activities, terms such as patient empowerment, patient participation, patient collaboration and patient engagement are interchangeably used [11, 12]. In research, involvement can occur at all different stages including during agenda setting, writing the research design, during the research, and during evaluation [11]. In this article, we define patient involvement as the meaningful involvement of patients and/or their loved ones, in one or more stages of scientific research.

**Fig. 2:**
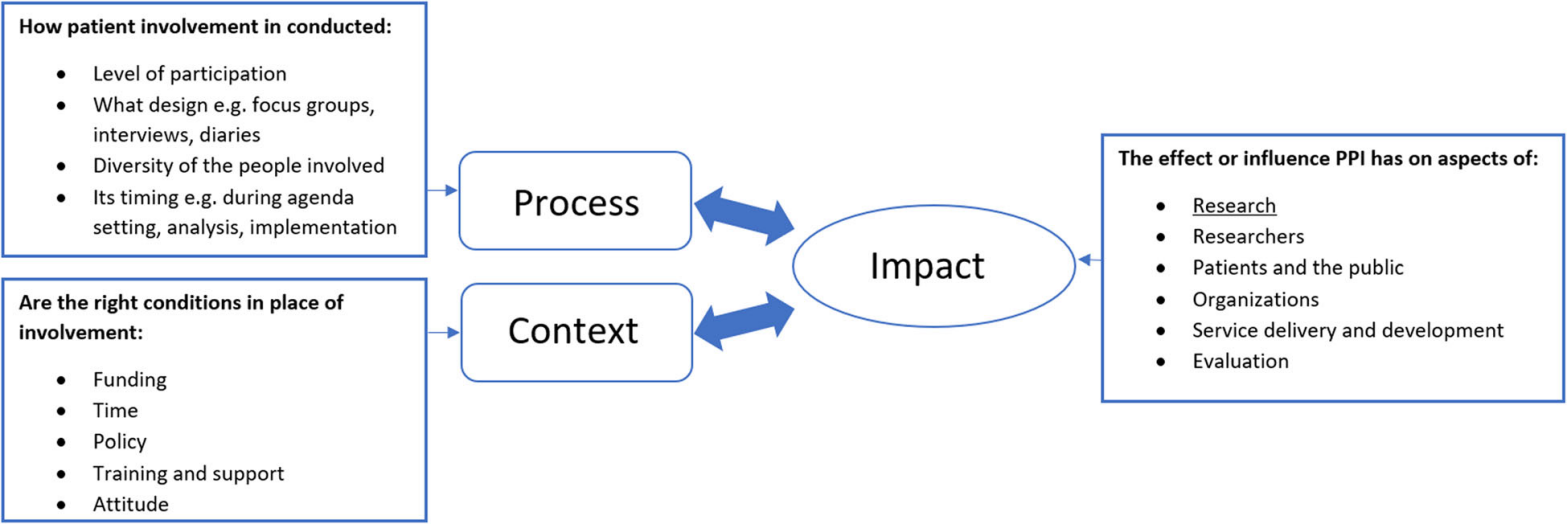
Impact evaluation framework for public and patient involvement (PPI). Based on the framework of Brett et al. [13]

The impact framework for public and patient involvement (PPI) by Brett and colleagues [13] was used to guide the analysis, taking into account that the process and context of the involvement influence its impact (Fig. 2). The **process** of patient involvement refers to specific aspects of the methodology and execution, including the timing of PPI, by which design, the level of involvement, and the diversity of the people involved. Diversity is particularly relevant, as patients are not a homogeneous group. Different studies require different patient subgroups, which might subsequently require different involvement approaches and methods [14]. In this study, the aspect ‘level of involvement ’ was not interpreted as ‘the higher the better’, but along a horizontal scale. This was a response to increasing criticism on the linearity of the participation ladder of Arnstein [15], which supposes higher levels to be better, instead of considering what is meaningful within a specific situation [16, 17]. The **context** of PPI refers to the conditions needed for involvement, including adequate funding and time, policy, adequate training and support, and a positive attitude towards PPI [3, 6, 13].

The format of the Guidance for Reporting Involvement of Patients and the Public (GRIPP2) was consulted during the study (See additional file) [18]. Our study was an evaluation of a PPI activity rather than a PPI study itself. However, most items on the list are applicable when looking at the content of the article. For example, we do (7a) report the results of the PPI which we studied, including both positive and negative outcomes. We (8d) also do comment on the ways in which the study contributes to the theoretical development of PPI. We found the GRIPP2 useful when writing the article, to ensure that all the important aspects of the studied PPI were reported.

## Methods

An exploratory qualitative research was conducted in 2017, using document analysis, semi-structured interviews and a focus group discussion [19–21]. In an early phase of the research, members of the patient committee were asked whether they would participate in the proposed methodology and suggest adaptations. There appeared to be no need for adapting the research design.

### Document analysis

Four types of documents were analysed: 1) the draft grant proposal (*n* = 14); 2) the written advice provided by members of the committee (n = 14); 3) the final grant proposal, as submitted for funding (n = 14); and 4), if applicable, the researcher’s reaction (i.e. rebuttal) on the provided advice (*n* = 7). The draft and final grant proposals were compared in order to analyse whether and how the advice had been incorporated into the final proposal. In addition, by analysing the written advice documents, themes that dominated the committee’s feedback were identified, in order to examine the relation between the advice that was provided and that which was incorporated [21].

Only submitted proposals written after 2016 were included in the analysis. Ongoing submissions were excluded. Proposals written before 2016 were excluded, in order to prevent recall bias in the subsequent interviews with researchers on how they processed the advice. Whether or not the proposal of a researcher was honoured was no selection criterium. In total 14 cases were included in the document analysis. Fundamental studies (*n* = 2), applied studies (*n* = 11) and implementation projects (*n* = 1) were covered, by researchers from nine different research institutes in the Netherlands. Documents were coded using open and thematic coding [19]. In order to increase the internal validity of the research, two documents were independently coded by two researchers from the project team (EV and IS) and discussed until consensus was reached [20].

### Semi-structured interviews

All main applicants corresponding to the 14 analysed cases were invited for an interview. Ten of them accepted the invitation (Table 1). The proposals discussed were for fundamental research (*n* = 2) and applied research (*n* = 7); one implementation project was discussed. For the majority of the researchers, their experience with patient involvement was limited to the committee’s advice. Four researchers already had some experience with patient involvement in a different phase of research, namely in the generation of research ideas, or in the involvement of patients in the communication of research results.

**Table 1:** Main characteristics of the participating researchers at the time of the research

	Male/female	Employed at	Research degree
Researchers	5/5	All at academic hospitals	8 post-docs, 2 professors

A topic guide, based on both the conceptual framework and findings of the document analysis, supported the interviews throughout [19–21]. The core topics were: researchers’ perception on asking for, receiving, and incorporating the advice; the usefulness and quality of the advice; and their ideas to improve the impact of the advice. Interviews were conducted in person, audio-recorded and transcribed verbatim. For one interview no permission was obtained for recording, therefore the extensive notes taken during this interview were used for analysis. Interviewees were sent a summary of the interview to confirm that it properly reflected their views and experiences. Transcripts were coded with a coding sheet based on the conceptual model (Fig. 2). In order to increase the validity of the coding process, the first two interviews were coded independently by two researchers of the project team (EV and IS) and discussed until consensus was reached [20].

### Focus group discussion

All members of the committee were invited to participate in a focus group discussion. Six members accepted the invitation. Due to medical issues, one member was not able to attend, but her previously e-mailed input was considered during the discussion. Table 2 illustrates the main characteristics of the participants.

**Table 2:** Main characteristics of participating committee members at the time of the research

	Male/female	Age	Duration membership committee	Employment status	Disease
Committee members	2/4	36–69 (μ53)	1 to 3.5 years	3 employed 2 unemployed 1 retired	Endocarditis, arteriosclerosis, myocardial infarction, congenital heart disease, stroke, aortic aneurysm.

Members were invited to share their experiences of the process and context of providing advice. During the focus group, they brainstormed about increasing the impact of their advice as well. A detailed script addressing relevant topics provided guidance during the focus group [19–21]. The focus group was audiotaped and transcribed verbatim. All participants received a summary of the discussion for a member check. A coding sheet, based on the conceptual framework and preliminary findings of the document analysis and interviews, was used during analysis.

## Results

The three main concepts of the Impact Framework by Brett et al. [13] – impact, process and context of patient involvement – were used to structure the results section and will be subsequently described below. We first focus on the impact of the patient committee’s advice on the content of grant proposals. Next, we report findings on the process, and finally we turn to reporting findings on the context of PPI.

### I. The impact of involvement

Document analysis on the 14 cases showed that in total 238 points of advice were given. Seven main topics could be distinguished on which the committee gave advice. Arranged in descending order, from the most recurring to the least: 1) Methodology (e.g. questions about inclusion/exclusion criteria for research participants, informed consent, interventions); 2) Communication with research participants, other healthcare professionals and communication of the research results; 3) Safety for the research participants in the study (e.g. privacy or side effects of treatment); 4) Understandability of the proposal (e.g. difficult terminology or unclear summaries); 5) Comments on the role of patients during the study, 6) Uncertainties about the background of the study (e.g. questions about the relevance of the study); and 7) Applicability of the research (e.g. future implications of the study). For each topic, Table 3 illustrates several examples of comments and advice the committee provided.

**Table 3:** Examples of advice given by the committee, for each of the seven main topics

Topic	Examples of advice given by the patient committee
1) Methodology	• “What are your selection criteria? What is your target group and when does a patient belong to this group?” • “A possible problem might be that the excretion of urine cannot be measured properly, as patients usually do not have a catheter.” • “Comatose patients cannot give their consent to participate. How will you inform the family or do they have to give their consent?”
2) Communication	• “Results of the study will be shared in several ways. Don’t forget the general practitioner in this, as that’s where the first signalling [of risks for disease] occurs.” • “It’s unclear if and how patients are informed about treatment options, and if they’re offered the chance to reject the treatment” • “It’s unclear how the research results will be shared afterwards with the patients who participated. Will they ever know in which group they were placed?”
3) Safety	• “Where will the data be stored?” • “Description of possible risks is lacking. If there are any risks, patients should be informed about it before they decide to participate in the study” • “The risks of undergoing several MRI scans in a short period of time is unclearly described. Also, it’s unclear to me how many MRI scans will be needed.”
4) Understandability	• “It’s a pity that the summary is in English [rather than Dutch]. It’s doable, but I had to google some abbreviations.” • “Too much medical terminology, e.g. renal, vasodilatation, diuresis etc. [...] more empathy is needed when you ask a lay man to think along.”
5) Patients’ role	• “How are patients exactly involved? It says that they are involved, but not how.” • “Collaboration with Harteraad is described, but it is unclear how they can influence the proposal. In other words: we can say a lot, but are you able to do something with it as well?”
6) Background	• “I miss information on the benefits of Shared Decision Making (SDM) and what currently goes wrong. [...] How many lives could be saved with SDM and how will it improve the Quality of Life of patients?” • “I miss the expected results and benefits [of the study] for patients. Also, I miss literature and numbers on the successes for existing interventions and how integration of these existing interventions with those in this study will lead to better results.”
7) Applicability	• “What will happen when risk factors can be recognized earlier, and who is going to do something with that information? Does the general practitioner have time to use such a diagnostic system? For patients it’s only useful if it involves risk factors you can do something about yourself” • “You assume that all Dutch hospitals will implement your findings, provided that the results of the study are positive. However, you’re not anticipating on a situation in which hospitals don’t want to follow your implementation plans.”

The committee’s advice stimulated researchers to change or sharpen their proposals. We noticed, for instance, the following changes after receiving the committee’s advice (see Table 4 for illustrating examples): 1) Added details on patient involvement throughout the research, 2) Use of simpler language, 3) Added information on the dissemination of research results among relevant stakeholders, 4) Added information on the risks and safety of an intervention, 5) Added details on relevance of the study.

**Table 4:** Examples of how grant proposals changed after receiving the committee’s advice

	Before the advice	After the advice
1) Added details on patient involvement	“During the preparation phase of this project proposal, the committee of experienced patients (Harteraad) was actively involved.”	“During the preparation phase of this project proposal, the committee of experienced patients (Harteraad) was actively involved. Patients reviewed a draft of the proposal and provided input. For example, an important comment involved privacy aspects. A discussion about privacy aspects of big-data analysis is considered important during the project. Therefore, we will set up a discussion panel, including researchers, doctors, legal officers and patients”
2) Changed use of language	Only an English summary	Dutch summary was added
3) Added details on dissemination of results	No information on dissemination of the research results	“The study outcomes will be shared with all relevant parties and stakeholders involved in [clinical] guideline adaptations, e.g., health care insurance companies, and determine the steps towards integration in guidelines.”
4) Added details on safety and risks of an intervention	1) Patient risks were not specifically mentioned. 2) No information on data storage	1) “Patient risks include the risk of hypernatremia, worsening heart failure and worsening renal function, which will be closely monitored during the study by adverse event monitoring.” 2) “Data will be stored in a protected database (Open Clinica)”
5) Added details on relevance of the study	“Shared Decision Making (SDM) is of great importance when decisions can have large consequences for quality of life (QoL).”	“SDM is of great importance when decisions can have large consequences for QoL. Research shows a weak, but positive relation between SDM and quality of life. When patients feel involved in decisions, it relates to a better understanding of possible choices, more satisfaction of the decision process and more trust in the physician.”

Analysis showed that 95 out of the 238 points of advice were incorporated in the submitted grant proposals and 143 were not. Considering the 143 unincorporated points of advice, which covered all abovementioned topics, interviewees mentioned that an unincorporated advice is not necessarily put aside. For example, document analysis showed that the following feedback was not incorporated in the final grant proposal:

“*The information section for hospitals [in the proposal] states that a one-cubic-centimetre- biopsy will be taken out of muscle tissue. Is that really necessary for this study or might less tissue be sufficient? What is the burden for the patient of this biopsy*?”

During the interview, the researcher who received this advice explained:

“*A remark on the size of a muscle biopsy will never be incorporated into a grant proposal because there’s not enough room for details [ …*] *The moment the research is executed and patient communication and informed consent letters are formulated, I do have space to explain these kinds of details to patients.*” (Researcher7).

The majority of researchers described similar situations, where due to word limits they felt they were not able to incorporate the feedback. They expressed their intentions to incorporate it in a later stage of the research. However, it was unclear whether incorporating feedback in a later stage would consist of providing additional explanations or modifying the research.

Analysis showed that it is not necessarily the topic of the advice that influences its incorporation into the final grant proposal, but the extent of its consequences. Recommendations for more explanation such as *“It’s not clear why...”, “It is not described how...”* are incorporated more often than comments disapproving the fundaments of the research, such as, *“I think this intervention isn’t a good idea.”* This shows that the impact of the provided advice is primarily on improving and refocusing the existing research idea and its execution. Several researchers confirmed this finding during the interviews: “*[Changing the whole method?] No that probably won’t happen, since in this phase you’re already very far with it [the proposal]”* (Researcher3). According to several interviewees, the input of patients should not be primarily on the method and analysis of the research, since they consider that their own expertise. Researchers differ in their flexibility and willingness to consider patients’ advice on the method section: *“I’d like to keep the choices for my research design and the analysis to myself.”* (Researcher15), and “*I was quite offended when patients commented on my power analysis [...] I know how to do a proper power analysis*. (Researcher14). Other researchers were more open for feedback on the methods and said to combine the patients’ input on methodological issues with the expertise of professionals and scientists*.* Most researchers suggested that the focus of the committee should be on the relevance, applicability and feasibility of the proposal and advice on distribution of the research results in the end. This is where, according to the researchers, the patient experiences are most valuable. The committee’s perspective on this issue was not discussed in detail, one member explained the following: “*I skip large pieces which are not interesting for me, such as statistical methods. I only scan them”* (committee Member2), illustrating that patients also do not consider this as their main expertise.

### II. The process of patient involvement

According to Brett et al. the process of patient involvement refers to specific aspects of the methodology and execution. Findings on the ‘design’, timing’, ‘level of involvement’, and ‘diversity of the people involved’ (Fig. 2) will be subsequently described.

### Design of involvement

There was a great diversity of opinions among researchers in terms of what, and which activities they perceived as patient involvement. For one researcher, patient involvement was *“Discovering whether your research meets the patient’s expectations*” (Researcher6). Most others were more specific and mentioned examples such as: involvement in generating research ideas; giving input on patient information letters and informed consent letters; taking part in a steering committee during the research; and assisting with the distribution of research results. These examples illustrate that according to researchers, involvement can be organized in different stages of research and can have different designs.

When discussing possibilities to strengthen the committee’s impact, changing the design of the PPI activity was offered as a suggestion. Several researchers suggested to pitch their research idea and/or proposal to members of the committee instead, or in addition to, the written feedback they currently receive. “*If you can pitch your ideas to an expert panel and they have the obligation of confidentiality and immediately give good feedback … that’s fantastic!*” (Researcher15). Interviewees mentioned that entering into dialogue with patients about their research proposal would be time-saving, as researchers would not have to wait for the committee’s written advice. Moreover, dialogue allows for more in-depth advice and quick responses to questions.

Furthermore, such a dialogue will be better accessible for a more diverse group, such as non-English speaking people and people without a scientific background: *“They [people with low health literacy] are not going to read the long complex pieces of text (...) Therefore I think a brainstorm session where they can provide immediate feedback would be great.”* (Researcher15). Several members of the committee prefer personal contact as well: *“I’d appreciate personal contact, whether it’s in person or over the phone. Having personal contact and checking whether my advice is understood correctly would be great*” (Committee Member4).

### Timing of patient involvement

The timing of the committee’s involvement – before submitting the grant proposal for funding – was positively perceived by several researchers: *“That was a really unique chance. I never experienced before that patients were already involved in writing the project idea”* (Researcher15). Two researchers suggested that a second round of feedback would be valuable, in order to get confirmation that they properly incorporated the committee’s feedback. Members of the committee similarly wished to have confirmation that researchers interpreted and acted upon their advice as they intended it to be, as not all researchers sent a rebuttal afterwards. Three researchers explicitly stressed that the advice may be more valuable in a later phase of the study, since patients can then help with the development and execution of the research. Financially, a later stage was also considered beneficial since after grant submission researchers have the budget to organize and finance involvement.

### Level of patient involvement

The amount of responsibility patients should or could have in research, was a complex issue according to both researchers and members of the committee. Almost all researchers said that there is a limit to this responsibility. Some said that making a decision on allocating funds was a bridge too far:

*“Our proposal was rejected by patient decision, and in my opinion that was undeserved. I sometimes really have the feeling that patients make a decision based on their own situation and feelings, instead of on scientific proof. So I don’t think patients should make the decision for allocating funding.”* (Researcher15).

To enlarge the impact of the committee members’ advice, some researchers suggested the opportunity to give more weight to the patients’ advice. Currently, the committee’s advice is noncommittal: *“You can put it aside completely [without consequences]”* (Researcher7). A possible solution to make the advice more compelling was offered during one of the interviews: *“I can imagine that when you send the advice along with the proposal [to the funding body], it’ll feel more binding and you’ll be forced to comment on the advice one by one.”* (Researcher7). This way, funding bodies will have better insight into the patients’ advice, and the reason it was or was not incorporated by the researcher into the final proposal.

### Diversity of people involved

Several suggestions were made by the researchers to increase the diversity of the committee. First, the committee could be enriched with relatives of patients, as they have another perspective and play an important role in providing proxy-consent. Second, considering the increased attention for prevention of cardiovascular diseases, one researcher suggested adding healthy people, citizens, (who, in theory, are all at risk of developing cardiovascular diseases), to the committee. Some interviewees had an ambiguous opinion about diversity in terms of patients’ educational level: *“On the one hand it is very good that they [patients] are able to read scientific proposals. But I am also curious what someone with a low social economic status might think of this.”* (Researcher15). Some interviewees mentioned examples of feeling misunderstood by the committee members, resulting in undeserved negative feedback. Although some of them blamed themselves for this misunderstanding (after all they wrote an unclear proposal), others questioned whether patients in general are equipped to provide constructive advice, let alone those without scientific training. Members of the committee confirmed that they sometimes struggle with fully understanding a proposal. How the proposal is delivered to the committee is an important facilitator or barrier for providing relevant advice. For example, focus group participants mentioned that the presence of a clear lay-summary in Dutch is truly essential for them, but such a summary is often lacking, incomplete or unclear.

### III. The context of patient involvement

This paragraph presents findings on the third concept of Brett’s model on PPI: its context. Context refers to the conditions needed for patient involvement to work. Findings on the ‘policy’, ‘funding’, ‘training and support’, ‘time’ and ‘attitude’ are subsequently addressed.

### Policy

Interviewees mentioned that funding bodies have an important and leading role in patient involvement in research. First, funding bodies increasingly oblige patient involvement. Second, their format requirements are guiding when writing a grant proposal.

*“If a funding body says: you should write this in your proposal [ … ] well then that’s what I do. Because the funding body asks for it. And that’s also the case for the component patient involvement. If they ask you to do it extensively then it should happen, otherwise, you don’t get funding at all. And if they say it’s not important, well then I don’t do it.“* (Researcher6).

Most researchers perceive the obligation of patient involvement as a novel, positive and logical movement while some others are very critical: “*It sometimes feels like an extra trick I have to perform [ …*] *meanwhile, I understand the trick and what I should write down to tick the box [of involvement*]” (Researcher9). In addition, the majority of researchers question whether involvement should be an obligation for all types of research. More than half of the researchers feel like patient involvement is less necessary or possible in fundamental research, arguing that it is an unknown territory for patients and difficult to identify with.

Changing the current policies was suggested to increase the impact of involvement. Two researchers observed that the feedback criteria of funding bodies, assessment committees and patient organizations were not aligned, which could lead to disappointment and rejection of funding. Therefore, one of them suggested that different parties involved in patient involvement in research, should align their assessment criteria and the expectations they have for patient involvement. Moreover, these two researchers preferred to have more guidance on how to organize patient involvement.

### Funding

Researchers mentioned that the issue of funding is of influence on how and when patient involvement is organized. It is necessary to cover the travelling expenses of patients, and it affects which design for patient involvement the researchers choose and in which moment of the research cycle. Before grant allocation, for example, there is no money available for organizing a focus group discussion with patients unless the research institute covers the expenses itself. Another participant suggested that the funding bodies could pay for it: *“If funding bodies find it important that patients are involved then they should incorporate it in the budget.”* (Researcher1).

### Training and support

Analysis showed that interviewees have multiple contradictory views about training and support of patient experts. On the one hand they are interested in a real layman’s opinion, but on the other hand they want members of the committee to know the basics of doing research, to have knowledge of grant application processes, and to understand that not every suggestion is possible within the timeframe and budget. Moreover, one researcher wanted them to have basic knowledge of human biology. In addition to training and support for themselves, members of the committee indicated that training and support for researchers could be an effective way to increase the impact of patient involvement activities. Focus group participants said that they regularly encounter prevailing misconceptions among researchers on patient involvement. “*It’s extremely important that patient involvement is already included in their curriculum*.” (Committee Member4).

### Time

Several researchers explained that generally the time available for writing a proposal is scarce and often takes place in their spare time, and therefore investing extra time in patient involvement can be difficult. One researcher explicitly stressed time as a barrier for optimal involvement. “*Ideally, I’d like to do much more on patient participation. Talk longer with patients, maybe even face to face or organize a meeting before I have a whole plan. [...] But in practice that’s unfortunately not possible.”* (Researcher6).

### Attitude

Although many researchers admitted that they organize patient involvement “because the funding body asks for it”, they do see the added value of including the patient’s voice. Interviewees especially valued the insights and ideas the committee contributed in terms of feasibility and relevance of the research. During the interviews, one researcher already expressed a change in his behaviour, namely that he contacted Harteraad for the second time in an earlier stage of his research to create more time for the committee to provide advice, and for himself to incorporate it. At the time of writing this paper, four out of ten interviewees contacted the committee again, suggesting that they were motivated by their earlier positive experiences with the committee.

Members of the committee recognize that the compulsory component of patient involvement has its downsides. They said they would like the committee to be wanted instead of being mandatory, as one of the focus group participants explained: “*For me the patient committee is a product we can put on the market: the market of scientists [ …]* *we need to present ourselves as being beneficial and an added value for the researcher.”* (Committee member 4).

## Discussion

The results show that the involvement of committee members helped researchers to rewrite and focus their proposals more in response to patients’ comments and suggestions. Although our study mainly focused on the impact of patient involvement on the research (proposal), the findings also indicate that our approach has an impact on the researchers involved. For example, researchers increased their knowledge and experience on patient involvement and were stimulated to increase the involvement of patients during their project. Also, researchers were more motivated to involve patients again at the start of a new project. Impact on patients is often described in terms of empowerment: by acknowledging their experiences and opinions, they learn to critically reflect, gain confidence and increase their knowledge on medical topics [13, 14, 22]. Although we recognise the impact that involvement has on patients, this was not measured within this study.

This study identified a number of aspects for strengthening the impact of patient involvement (either related to procedural or contextual issues) including: the timing of patient involvement (just before a deadline or during reflection), the design of the involvement (written or via a dialogue), diversity of the involved patients (e.g. education, daily activity, age, etc), the level of training of both researchers and patients, attitude towards PPI, and the policy of the funding agency.

### Timing and design of involvement

Advice requiring fundamental changes in the design of the research was often not incorporated. Modifying procedural or contextual aspects of the current involvement activity might create opportunities to enhance the impact and capture advice regarding design and methodologies as well. This is desirable since patient involvement can, for example, lead to more relevant and feasible outcome measures and interventions. In the current process of the patient committee, researchers often contact the committee near the submission deadline and little time is left to change major aspects of the proposal. Although some researchers wanted to keep methodological and analytical decisions mostly to themselves, involving the committee earlier in the designing and writing process could enable researchers to adapt more fundamental advice as well.

One interesting option is to explore the added value of researcher-patient/public dialogues, where researchers can pitch their research ideas to patients in an early stage of the research cycle, and get immediate feedback and brainstorm opportunities in return. Harteraad is currently experimenting with this approach in collaboration with several large research funding bodies in the Netherlands. The preliminary analysis of this approach shows that it leads to fruitful dialogues and an exchange of ideas. Using pitches and dialogues enables non-English speaking patients to become involved. Moreover, when researchers pitch their research in understandable language and create room for questions, people from a wider range of education can participate as well. However, it remains a challenge to make sure that different stakeholders speak the same language and are able to understand each other’s perspectives.

### Diversity

Researchers in this study asked for what they considered to be relevant and reasonable feedback, but simultaneously wanted advice from the ‘real layman’. Several researchers have suggested that one should be cautious of the protoprofessionalization of patients wherein they learn to think along with researchers, while losing their ‘pure’ patient experience [4, 23–26]. Ideally the process of PPI should accommodate all patients, from all groups of society. Considering the challenge it already is for trained members of the committee to provide advice, the current design by which Harteraad organizes involvement it not accessible for all groups of society, and one could raise doubts about whether the current design is possible without a certain extent of professionalization. This is in line with Van Reybrouck [27], who stated that laymen are able to valuably engage in a discussion, provided that they have relevant training and knowledge.

Attaining relevant and reasonable feedback from all groups in society, without too much professionalization, might be accomplished by looking for additional, more open and creative involvement designs. Members of the committee and researchers both agreed that the current design of reading English scientific grant proposals is not suitable for all groups in society. Sunwolf and Leets [28] also wrote that verbal communication is an important way to exclude patient representatives. When looking for alternatives to enlarge the diversity of involved patients, ask people of the (marginalized) groups one is looking for, how to reach out and enable these difficult-to-access participants [29]. It is important to note that diversity covers more aspects than level of education. Although it was not within the scope of this study, we experienced, for example, that the severity of one’s disease affects the energy available for participation and the amount of spare time a participant has available next to activities like a job and family care. Also, when the meetings are organised influences the possibility to participate (e.g. during working hours or in the weekend).

### Training

Different roles might require different skills and knowledge, and depending on the role and type of involvement, the need for training varies [23, 30]. As stated before, one should be cautious of patients losing their ‘pure’ patient experience [24]. However, when patients collaborate on a high level of involvement, knowing the basics principles of the research process, outcome measures, and having an understanding of qualitative and quantitative research methods can make it easier to contribute [22, 30]. Members of the committee receive similar information during their training, and find it helpful for providing advice as well. PPI experts are not univocal about whether or not patients should receive training in the first place, and if so, how much and what kind of training should be provided so as to maintain the ‘pure patient perspective’. Critics point to the risk of overshadowing of the lived experience of patients and the need to train researchers in how to accommodate patient perspectives and needs [23, 25, 26, 30, 31]. While others argue that training can lead to both better understanding of the research process with subsequently qualitatively better advice, and better understanding of the research language. The latter might improve the dialogue between researchers and patients. The patient does not need to become a researcher by all the training, but the training will help to become a better discussion partner and integrate the patient perspective within discussions [26, 30]. Discussion tools such as the Participation Matrix can be helpful for both patients and researchers to discuss different roles, and the need for training to be able to fulfil that role [32] .

For researchers, stimulating intrinsic motivation and cultural change should receive major attention, because it is the way to more sustainable and meaningful patient involvement [33]. One way to foster intrinsic motivation is by offering researchers training and support on both the added value of patient involvement and the practicalities to execute it. Many researchers worldwide lack the knowledge, skills and experiences on how to involve patients [34]. Patient ambassadors could play an important role in creating and executing this training and support [35]. Moreover, personally experiencing the added value of patient involvement is thought to induce positive perception towards it [33, 36]. In order to achieve intrinsic motivation of the researcher in the long run, patient involvement should become part of the academic curriculums of (at least) medicine, biomedical science and nursing. This will ensure that researchers are provided with the basics of patient involvement in their early career and are accustomed to the involvement of patients in their research [35, 37]. An example of how this could be organized is The Hague University of Applied Sciences, which integrated the course ‘participative healthcare’ in its curriculum for nurses. This course focusses on both the theory of patient involvement in healthcare, as well as the required practical skills. A similar course focussing on participation in research for (bio) medical students, could be an opportunity.

### Policies of funders

Findings of this research show that policies of funding bodies that increasingly oblige patient involvement, are an important motivator for researchers to contact Harteraad. This is in line with Elberse [10], who stated that funding bodies can create incentives for researchers by the incorporation of involvement as one of the conditions for funding. Moreover, acknowledging that involvement of patients costs money, enabling the incorporation of these costs in the budget sheet, is helpful. Providing directions for researchers on the website of the funding body is a stimulus to start patient involvement as well. Funding bodies could contribute to that, by providing easily accessible involvement guidelines for researchers, and by collaborating with patient organizations and other funding bodies on their expectations of involvement.

The PPI approach of Harteraad is organised in an early phase of the research cycle, in the designing phase. It would be of added value if patients have a role as co-assessor for grant requests as well, and we would like to recommend funding bodies to implement such an approach [26]. Similar to the current advice of the patient committee of Harteraad, patients would be able to assess the relevance, applicability and feasibility of the grant requests, alongside the scientific quality as assessed by a scientific committee. The Dutch Heart Foundation has already used such an approach of including the patient perspective in funding allocation.

Organizational change is needed to enhance the impact of patients’ advice as well. Funding bodies can insist on transparency from researchers on how they dealt with patient input and ask that they justify their choices. As researchers tend to adhere to funding bodies’ policies, this activates them to take patient input seriously and hopefully also experience its added value. For our case specifically, researchers should be asked to send the advice received from members of the committee and their rebuttal along with the grant proposal. Hereby, the advice will be less informal, and its impact will, most likely, be increased [38]. At the time of writing, the committee and Harteraad already have made efforts to implement this recommendation.

### Strengths and limitations

**Fig. 3:**
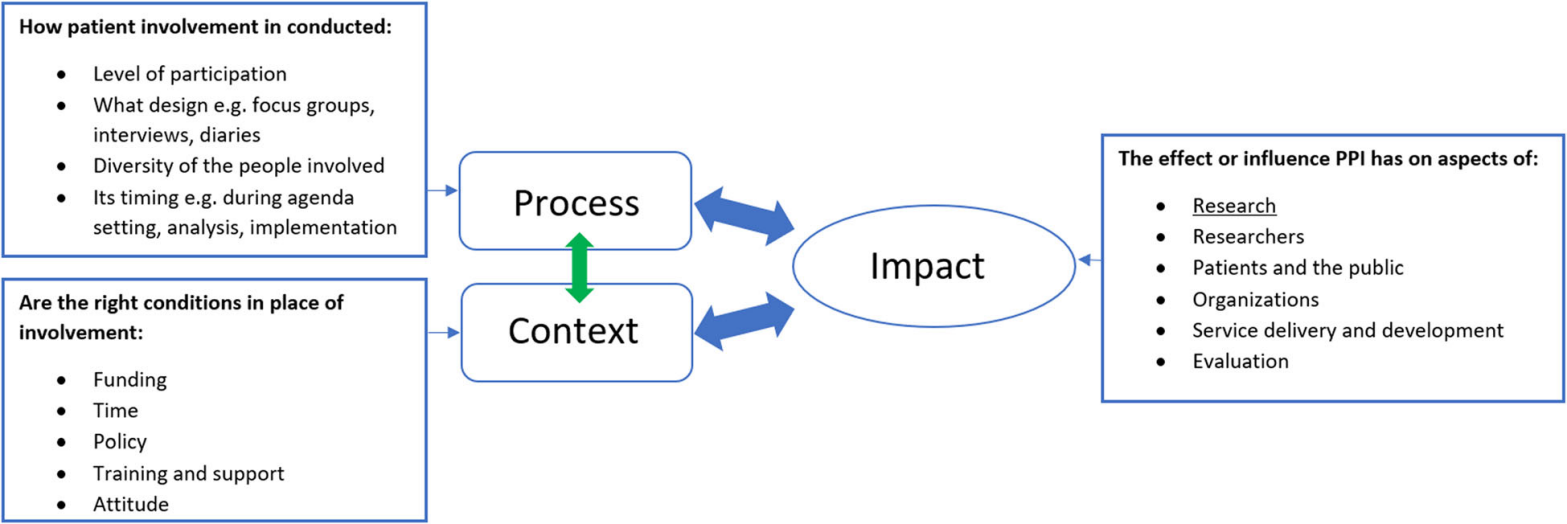
The influential relationship between process and context found in this research. Illustrated within the model of Brett et al. [13] by the green arrow

Brett’s model [13] proved to be a valuable tool for the analysis of this study; however, the relation between process and context could receive more attention (Fig. 3). For example, the funding (context) to facilitate involvement proved to be influential for the timing of organizing involvement (process).

This research made it possible to explore the direct impact of the committee’s advice on the submitted grant proposals. While the researchers indicated that some parts of the committee’s advice would be incorporated in a later phase of the study, it does not necessarily mean that they will act accordingly in practice. Research using a combination of interviews and participatory observation during actual execution of the research projects, will provide a more complete understanding of the impact of involvement.

Another limitation of this study is its scope on impact in terms of impact on research, rather than in the broader sense: on the researchers and patients involved. Although we did find aspects of the ‘intangible’ impact such as improved attitude for researchers, future research on the broader impact of PPI would be of added value.

Moreover, the relation between PPI and chances of funding allocation was beyond the scope of this study. As this would be interesting for researchers, patients and the public, as well as funding bodies, future research in this area would be recommended.

## Conclusion

This case study aimed to evaluate whether the written advice of a patient’s committee helped cardiovascular researchers to improve their proposal before submitting it for funding. Overall, we found that more than one third of the points of advice given by the patient committee were incorporated into the final proposals: researchers added details on the relevance and the risks of the study for patients, used simpler language and added information about the role of patients throughout the research. Procedural and contextual changes in the current process of the patient committee could enable researchers to adapt more fundamental suggestions as well. We believe that the lessons learnt are relevant for others organising patient involvement in research, particularly in projects in which patients have an advisory role. Further effort is required to increase the value that is currently attributed to patient involvement, and to support researchers in the required organizational and cultural changes to meaningfully involve patients in research.

## Data Availability

The dataset on the document analysis generated and analysed during the current study are not publicly available and cannot be requested, due to confidentiality of the included documents. The datasets on the interviews and focus group discussion generated and analysed during the current study are not publicly available due to promised anonymity, but are available (in Dutch) from the corresponding author on reasonable request.

## Abbreviations

GRIPP2: Guidance for Reporting Involvement of Patients and the Public 2
PPI: Public patient involvement
QoL: Quality of life
SDM: Shared decision making
